# Ritonavir-Loaded PEG-*b*-PCL Polymeric Micelles: Reduced Excipient Requirements and Comparable Oral Pharmacokinetics in Rats

**DOI:** 10.3390/ph19091491

**Published:** 2026-09-19

**Authors:** Eungyeop Lee, Jihoon Lee, So-Young Park, Hyunji Lee, Duhyeong Hwang, Dong Wuk Kim

**Affiliations:** 1 BK21 FOUR Community-Based Intelligent Novel Drug Discovery Education Unit, College of Pharmacy and Research Institute of Pharmaceutical Sciences, Kyungpook National University, Daegu 41566, Republic of Korea; sa03103@naver.com (E.L.); legadema0905@naver.com (J.L.); soyoung1021@knu.ac.kr (S.-Y.P.); 2 Vessel-Organ Interaction Research Center (VOICE, MRC), Kyungpook National University, Daegu 41566, Republic of Korea; 3 Mass Spectrometry Based Convergence Research Institute, Kyungpook National University, Daegu 41566, Republic of Korea; 4 College of Pharmacy, Kyungsung University, Busan 48434, Republic of Korea; hlee@ks.ac.kr; 5 College of Pharmacy, Keimyung University, Daegu 42601, Republic of Korea

**Keywords:** Ritonavir, HIV-1 protease inhibitor, PEG-*b*-PCL, polymeric micelle, lyophilization, oral pharmacokinetics

## Abstract

**Background:** Ritonavir (RTV), an HIV-1 (human immunodeficiency virus-1) protease inhibitor, has low aqueous solubility and poor oral bioavailability, requiring large amounts of excipients that result in bulky dosage forms and poor patient compliance. We aimed to develop an RTV-loaded polymeric micelle formulation with high drug loading capacity that improves aqueous solubility, reduces the excipient burden and associated excipient-related adverse effects, and remains stable after lyophilization without a cryoprotectant. **Methods:** RTV-loaded micelles were prepared from the amphiphilic block copolymer poly(ethylene glycol)-*block*-poly(ε-caprolactone) (PEG-*b*-PCL) by thin-film hydration. Particle size and the polydispersity index (PDI) were measured by dynamic light scattering and encapsulation efficiency and drug content by HPLC, before and after lyophilization. The drug-to-excipient ratio was compared with the commercial product (Norvir^®^), and pharmacokinetics were evaluated in male Sprague-Dawley rats after single oral administration. **Results:** Micelles showed a mean particle size of 25.9 ± 0.09 nm, a PDI of 0.224 ± 0.008, and an encapsulation efficiency of 98.9 ± 1.54%, with particle size and drug content retained after lyophilization and reconstitution without any cryoprotectant. Drug content reached 27.3% (*w*/*w*) versus 12.5% for the commercial product, a 2.6-fold reduction in excipients per unit dose. In rats, the micelle formulation showed pharmacokinetic parameters comparable to those of the Norvir^®^ suspension (C_max_, 2243.9 ± 1226.4 vs. 2106.1 ± 835.9 ng/mL; AUC_last_, 6695.3 ± 2162.6 vs. 8178.2 ± 2892.1 h·ng/mL; T_max_, 0.9 ± 0.6 vs. 1.5 ± 0.5 h), with no significant differences. **Conclusions:** The PEG-*b*-PCL micelle achieved high RTV loading, substantially reduced excipients, and provided oral exposure comparable to the commercial product, supporting its potential as an alternative oral delivery system.

## 1. Introduction

HIV (human immunodeficiency virus) remains one of the most critical global public health issues, with approximately 39.9 million people worldwide living with it in 2023 [1]. While a complete cure for HIV has yet to be discovered, antiretroviral therapy enables patients to achieve a life expectancy comparable to that of people without HIV [2]. Antiretroviral drugs are broadly classified based on how they interfere with the HIV life cycle, including nucleoside reverse transcriptase inhibitors (NRTIs), non-nucleoside reverse transcriptase inhibitors (NNRTIs), protease inhibitors (PIs), and integrase strand transfer inhibitors (INSTIs) [3]. HIV treatment regimens typically comprise a combination of two or three drugs from at least two distinct classes, an approach commonly referred to as combination antiretroviral therapy (ART) [4]. Many antiretroviral drugs, however, exhibit low aqueous solubility and are classified as BCS Class II or IV, resulting in poor oral bioavailability in humans [5]. Moreover, several antiretroviral drugs require high-dose administration with substantial amounts of excipients to facilitate efficient absorption, resulting in a high pill burden that reduces patient compliance and quality of life [6,7,8].

Polymeric micelle systems can play a key role in addressing these formulation challenges. Composed of amphiphilic block copolymers that self-assemble into core–shell structures above the critical micelle concentration (CMC), polymeric micelles physically encapsulate hydrophobic drugs within their core while the hydrophilic shell confers aqueous solubility and protects the cargo from oxidation, premature clearance, and immune recognition [9,10,11,12,13]. By tailoring the copolymer structure, these systems can be engineered for targeted delivery [14,15,16,17], controlled release [18], enhanced solubilization [19,20,21], and reduced toxicity [22,23,24,25]. Among the biocompatible polymers used for this purpose, poly(ethylene glycol) (PEG) is widely employed as the hydrophilic segment owing to its FDA-approved safety profile, excellent aqueous solubility, and anti-fouling properties that reduce protein adsorption and immunogenicity [26,27,28,29,30,31,32]. For the hydrophobic segment, poly(ester)s such as poly(ε-caprolactone) (PCL) are commonly used owing to their superior biodegradability, with hydrolytically cleavable ester linkages and an aliphatic domain capable of encapsulating insoluble small molecules [33,34,35,36,37].

Ritonavir (RTV) is an HIV-1 protease inhibitor with very low aqueous solubility and poor oral bioavailability, classified as a BCS Class II drug, and is commercially available under the marketed name Norvir^®^ [38]. A single tablet contains 100 mg of RTV in a total tablet weight of 800 mg, meaning that the drug accounts for only 12.5% (*w*/*w*) and is accompanied by approximately seven times its weight in excipients. With a recommended regimen of 600 mg twice daily, this necessarily results in a high daily excipient burden. Despite various formulation approaches explored to improve RTV solubility and reduce its excipient load, achieving high drug loading with a minimal carrier amount remains a challenge. In this study, we developed a novel RTV-loaded polymeric micelle using PEG-*b*-PCL copolymers to address these limitations. We synthesized PEG-*b*-PCL of different molecular weights and assessed the characteristics of each polymeric micelle. The optimized formulation was further studied for stability to lyophilization, in vitro drug release, and transmission electron microscopy (TEM) to assess the complete encapsulation of RTV and the morphology of RTV-loaded polymeric micelles. Lastly, an in vivo pharmacokinetic study and comparative analysis with the conventional formulation were conducted on rats by oral administration.

## 2. Results and Discussion

### 2.1. Characterization of PEG-b-PCL Copolymers

**Figure 1:**
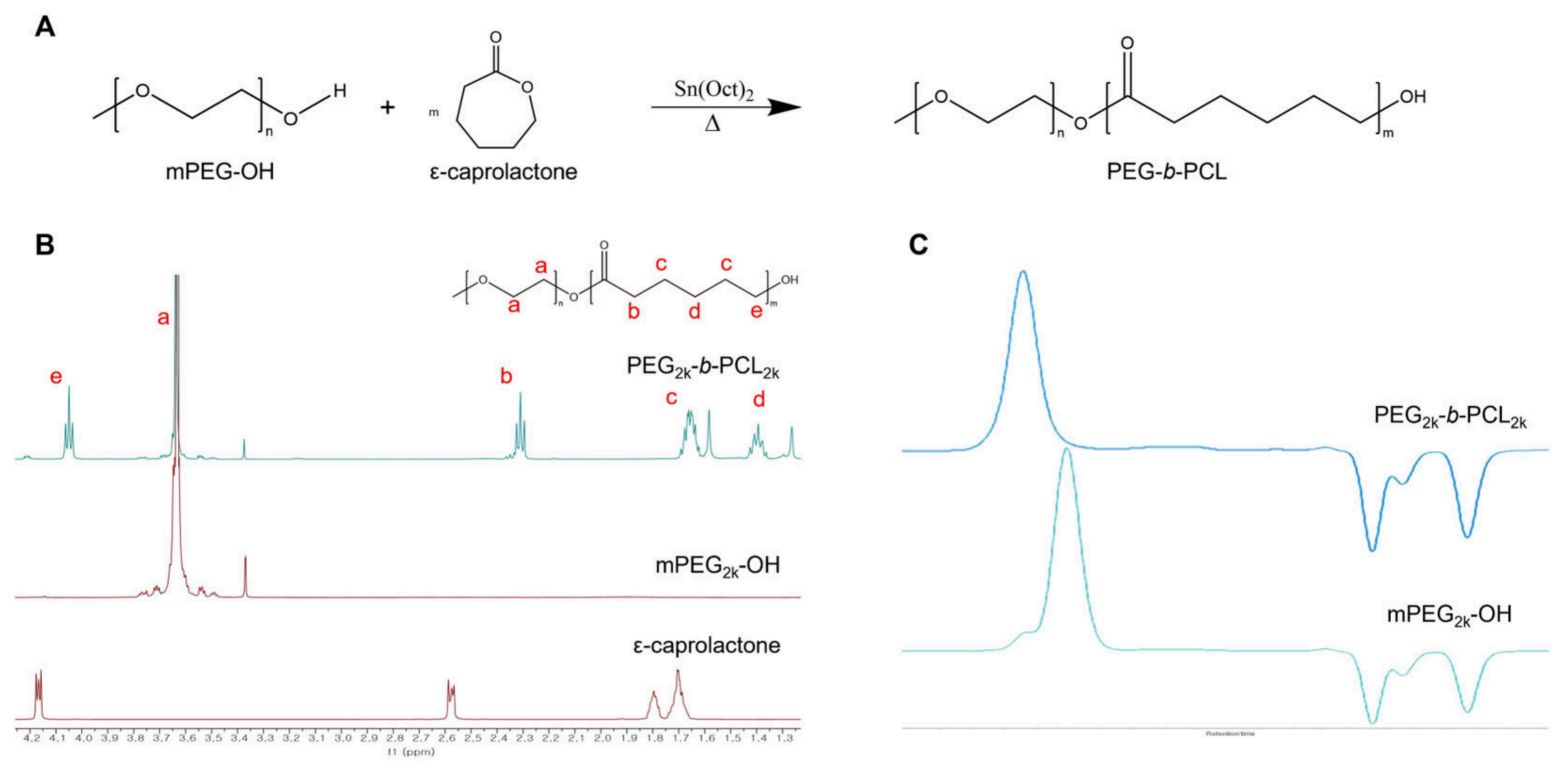
Synthesis and characterization of PEG-*b*-PCL copolymers. (**A**) Synthetic scheme. (**B**) ^1^H NMR spectra of mPEG_2k_-OH, ε-caprolactone and PEG_2k_-*b*-PCL_2k_ dissolved in CDCl_3_. (**C**) GPC chromatograms of mPEG_2k_-OH and PEG_2k_-*b*-PCL_2k_.

We synthesized PEG-*b*-PCL copolymers with varying combinations of PEG and PCL block molecular weights to investigate how the characteristics of drug-loaded polymeric micelles are influenced by differences in the molecular weights of each constituent block. We used two different molecular weights of mPEG-OH (MW = 2000 and 4000 g/mol) as initiators of ring-opening polymerization (ROP). The feed ratios of caprolactone were calculated to obtain three different molecular weights of the PCL block targeting 1500, 2000 and 2500 g/mol. The synthetic scheme of the PEG-*b*-PCL copolymer, ^1^H nuclear magnetic resonance (^1^H NMR) spectrum, and gel permeation chromatography (GPC) chromatogram of the synthesized PEG-*b*-PCL copolymer are shown in Figure 1. The peak at δ = 3.64 ppm was assigned to the methylene protons (-CH_2_) from the PEG block, and the peaks at δ = 1.38, 1.65, 2.31 and 4.06 ppm were assigned to the methylene protons from the PCL block (Figure 1B) [39,40]. The number-average molecular weight (M_n_) of each copolymer was determined by ^1^H NMR based on the integration ratio of the characteristic peaks of the PEG and PCL blocks, which provides an absolute measure of the block composition. In addition, GPC was performed using DMF as the eluent, employed as a complementary technique to evaluate the relative molecular weights (M_n_ and weight-average molecular weight, Mw) and the polydispersity index (PDI), which reflects the uniformity of the molecular weight distribution and cannot be obtained by ^1^H NMR alone. As shown in the unimodal peak of GPC chromatograms (Figure 1C), PEG-*b*-PCL copolymers were successfully synthesized with a narrow PDI, indicating a narrow molecular weight distribution of the copolymer chains. The M_n_ values obtained by GPC were higher than those determined by ^1^H NMR, which is attributed to the use of polystyrene standards for GPC calibration, whose hydrodynamic volume differs from that of PEG-*b*-PCL copolymers. The molecular weight characteristics (M_n_, M_w_, and PDI) of PEG-*b*-PCL copolymers determined by ^1^H NMR and GPC are summarized in Table 1.

### 2.2. Characterization of RTV-Loaded Polymeric Micelles

The physicochemical characteristics of RTV-loaded polymeric micelles prepared with different molecular weights of synthesized PEG-*b*-PCL are summarized in Table 2. The micelles prepared with PEG_2k_-*b*-PCL_1.5k_ and PEG_2k_-*b*-PCL_2k_ had the largest mean particle size of 91.3 ± 2.24 nm and the smallest of 26.3 ± 0.19 nm, respectively. The micelles prepared with PEG_2k_-*b*-PCL_2k_ and PEG_4k_-*b*-PCL_2k_ had the highest encapsulation efficiency of 96.4 ± 0.79% and the lowest of 31.7 ± 0.65%, respectively. In addition, the micelles prepared with PEG_2k_-*b*-PCL_2k_ had the narrowest PDI of 0.130 ± 0.032, indicating its uniform size. Student’s *t*-test confirmed that all measured parameters of PEG_2k_-*b*-PCL_2k_ micelles were significantly different from those of all other copolymer compositions (*p* < 0.05). Considering the results, we concluded that PEG_2k_-*b*-PCL_2k_ was the optimal copolymer for RTV-loaded polymeric micelles. These trends likely reflect the hydrophilic/hydrophobic balance of the copolymers. PEG_2k_-*b*-PCL_2k_ provides an optimal core volume and PEG corona thickness for efficient chain packing and RTV–PCL hydrophobic interaction, whereas longer PEG (4k) increases corona thickness and CMC, reducing effective core capacity, and PCL_2.5k_ likely disrupts core packing, both lowering drug loading.

**Figure 2:**
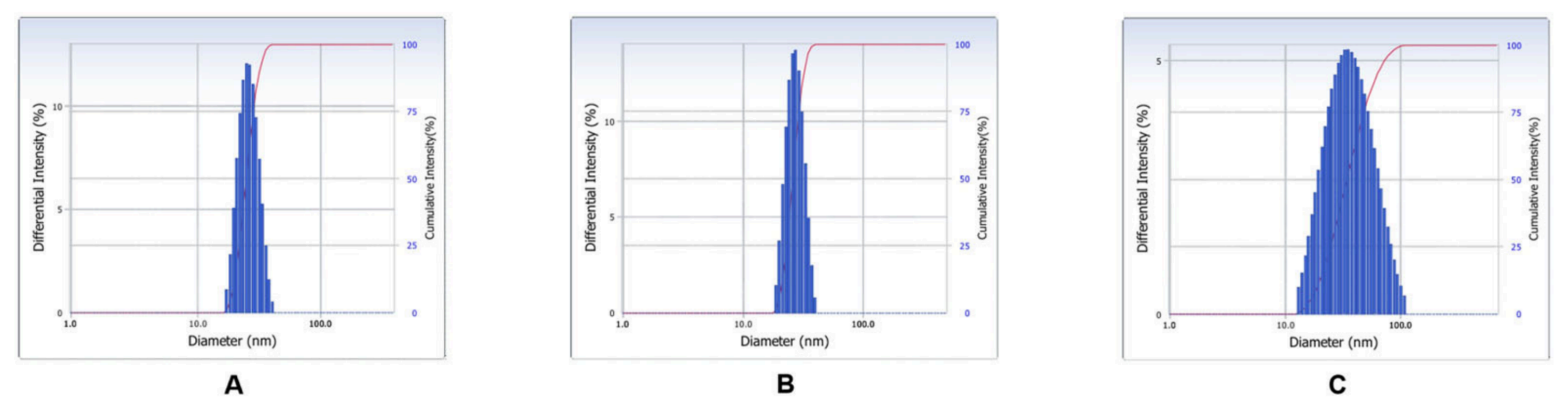
Particle size distribution of drug-loaded polymeric micelles prepared in different solvents; (**A**) acetonitrile, (**B**) methanol and (**C**) ethanol.

To evaluate the influence of organic solvents on drug-loaded film formation, micelles were formulated using three different organic solvents. All micelle formulations achieved high drug loading efficiencies exceeding 90% with a unimodal size distribution and narrow PDI across tested solvents (Figure 2 and Table 3). These results indicate that the drug and polymer were effectively mixed and molecularly dispersed within the resulting films after the evaporation of the organic solvent tested. Notably, the micelles prepared in methanol exhibited the smallest mean particle size of 25.8 ± 0.99 nm and the highest drug loading efficiency of 93.9 ± 3.08%; thus methanol was selected as the optimal solvent for constructing drug-loaded polymeric micelles. This may be attributed to methanol’s higher polarity and water miscibility, which likely promoted faster polymer precipitation and more efficient RTV encapsulation. Residual methanol in the final formulation was not quantitatively assessed and should be evaluated in future studies.

**Figure 3:**
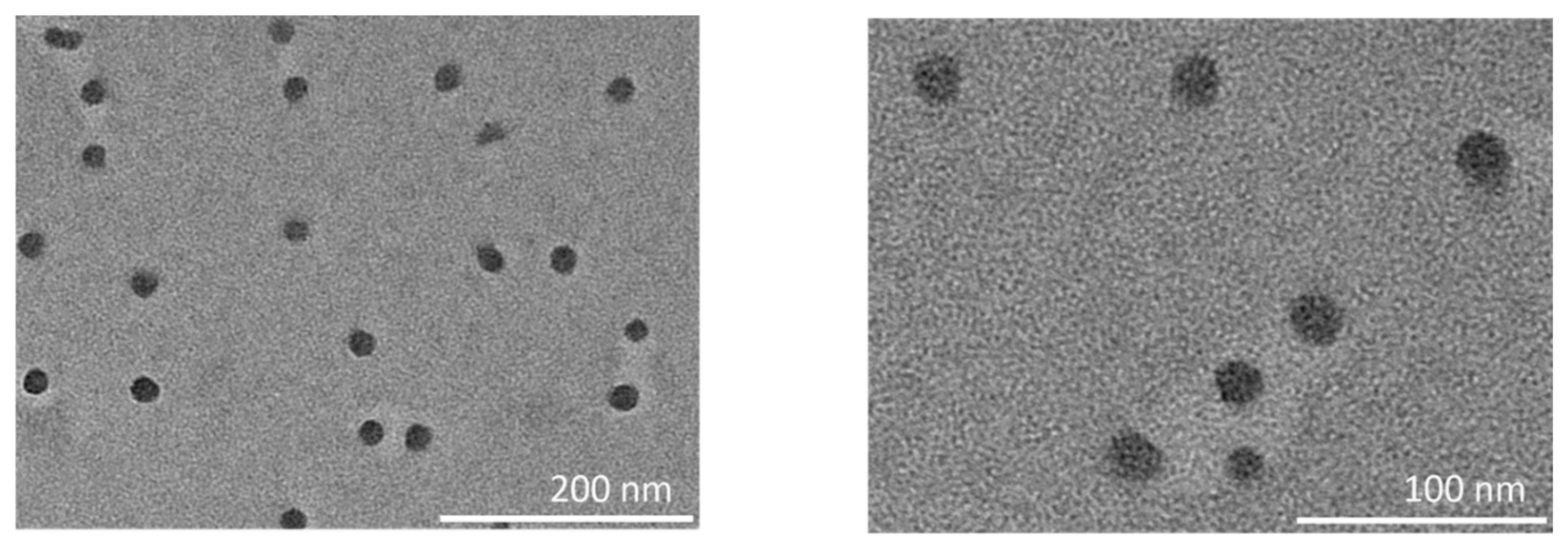
Morphology of RTV-loaded polymeric micelles analyzed by TEM (stained with 1% sodium phosphotungstate).

In the assessment of micelle morphology, TEM images revealed that RTV-loaded polymeric micelles exhibited a predominantly uniform spherical shape (Figure 3). The DLS results indicated a narrow hydrodynamic size distribution in aqueous solution, with diameters of approximately 20 to 30 nm, while TEM provided direct morphological evidence of the spherical structures. It should be noted that the drying process required for TEM sample preparation may cause the shrinkage of the soft polymeric micelle structure, and thus the TEM diameter may not directly reflect the hydrodynamic diameter measured by DLS.

### 2.3. Stability to Lyophilization

**Figure 4:**
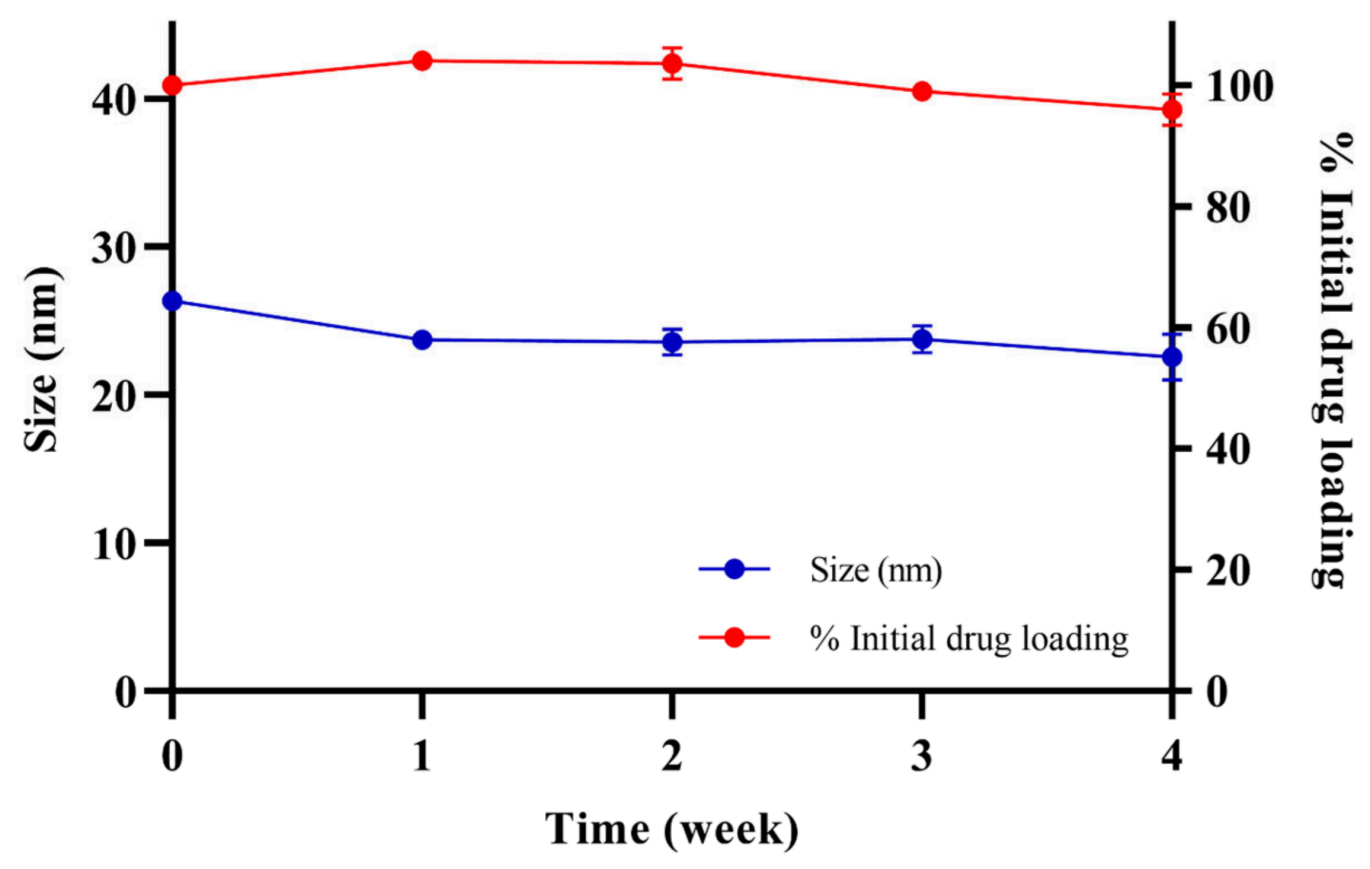
Changes in particle size and drug loading content after reconstitution of lyophilized micelle solution stored under room temperature over time.

Since polymeric micelle formulations are preferred as a lyophilized powder to improve the stability of the formulation and maintain drug loading, we assessed the stability of RTV-loaded polymeric micelle to lyophilization in terms of drug loading and size distribution. After lyophilization and subsequent reconstitution with aqueous media, the RTV-loaded polymeric micelles retained their original physicochemical properties (Table 4). The lyophilized powder appeared as a white, porous cake with no visible collapse or discoloration and reconstituted rapidly (within seconds) upon the addition of aqueous media. Residual moisture content and recovery percentage were not quantitatively assessed, although no visible signs of physical degradation were observed. Specifically, the particle size distribution remained consistent at 24.4 ± 0.45 nm, and the drug loading efficiency was maintained at 100.2 ± 1.48%, a value considered equivalent to ~100% within the analytical variability in HPLC quantification. To evaluate the short-term (4-week) stability of the RTV-loaded polymeric micelle formulation, lyophilized micelle powders were stored under room temperature for various durations, after which the drug content and size distribution of the formulations were analyzed. Following reconstitution, the micelle formulations maintained consistent particle sizes ranging from 20 to 30 nm and demonstrated drug loading efficiencies exceeding 90% (Figure 4), indicating decent stability upon lyophilization. This cryoprotectant-free stability may be attributed to the dense PEG corona surrounding the micelle core, which provides steric stabilization against aggregation during freezing and dehydration, functioning analogously to conventional lyoprotectants such as trehalose or mannitol.

### 2.4. In Vitro Drug Release Study

**Figure 5:**
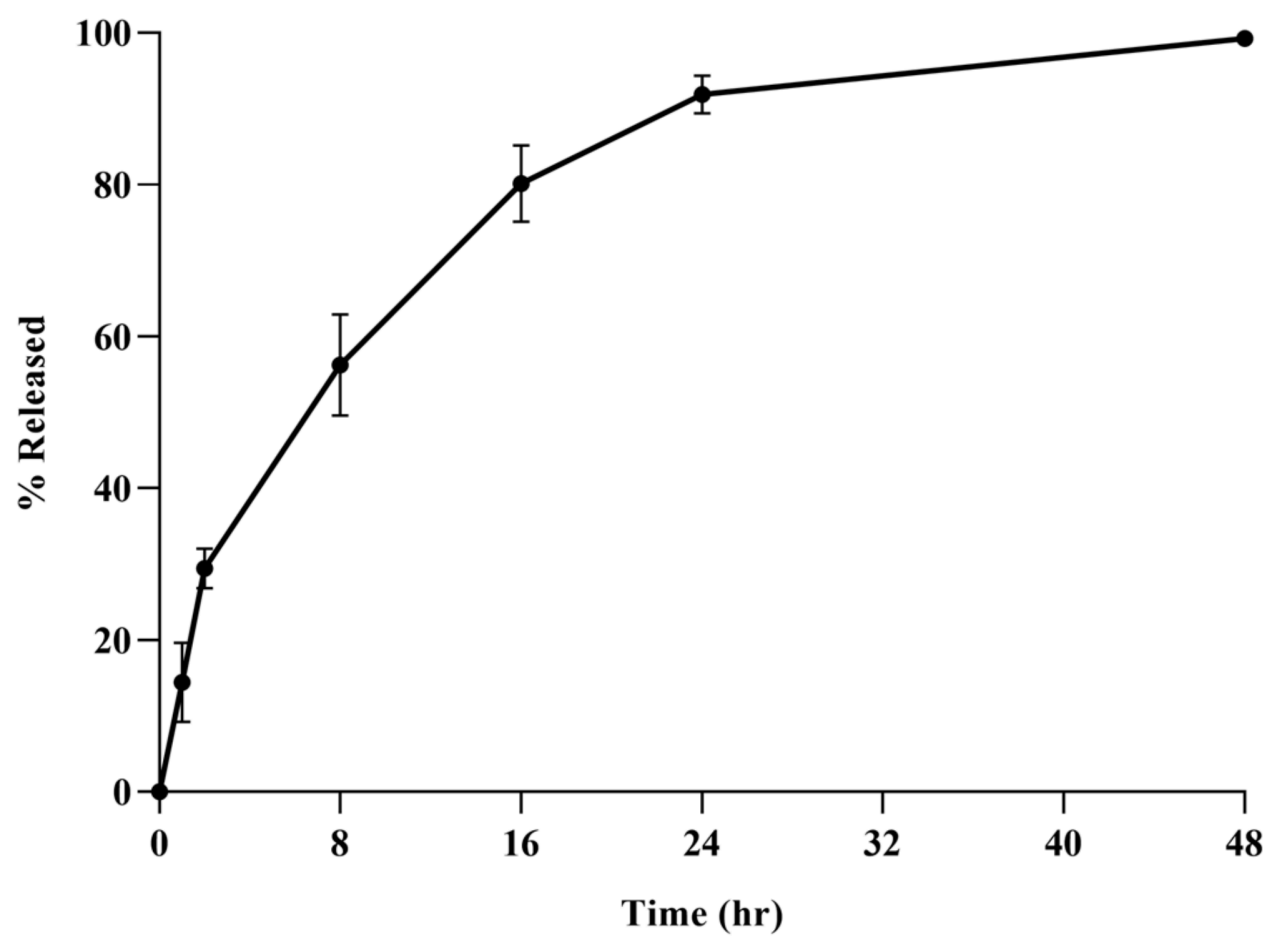
Drug release from drug-loaded polymeric micelles incubated in pH 1.2 at 37 °C over time.

The in vitro drug release of RTV-loaded polymeric micelles was evaluated using a pH 1.2 solution to simulate gastric conditions following oral administration (Figure 5). This acidic environment was chosen to mimic the gastric fluid conditions encountered after drug administration, thereby assessing the drug release performance of RTV-loaded polymeric micelles during the early stage of gastrointestinal transit. Although the physiological gastric residence time is generally 0.5–4 h, the release study was extended to 48 h to fully characterize the intrinsic release kinetics of the micelles rather than to simulate gastric transit time itself. RTV was continuously released from the micelles under pH 1.2 media at 37 °C with approximately 90% released within 24 h and almost completed after 48 h. The cumulative release profile demonstrated prolonged RTV release under the acidic in vitro conditions examined, with an initial rapid release phase observed within the first 6 h, followed by a slower, steady release up to 48 h. Release behavior under intestinal conditions (pH 6.8–7.4, with bile salts or enzymes) was not evaluated in this study, and further investigation under such conditions would provide a more complete understanding of the in vivo release profile of this formulation. In addition, the release data were not fitted to kinetic models, which would help elucidate the underlying release mechanism and is planned for future work. A free-drug dialysis control was not included, and thus membrane diffusion cannot be fully excluded as a contributing factor to the observed release.

### 2.5. Comparative Pharmacokinetic Study in Rats

**Figure 6:**
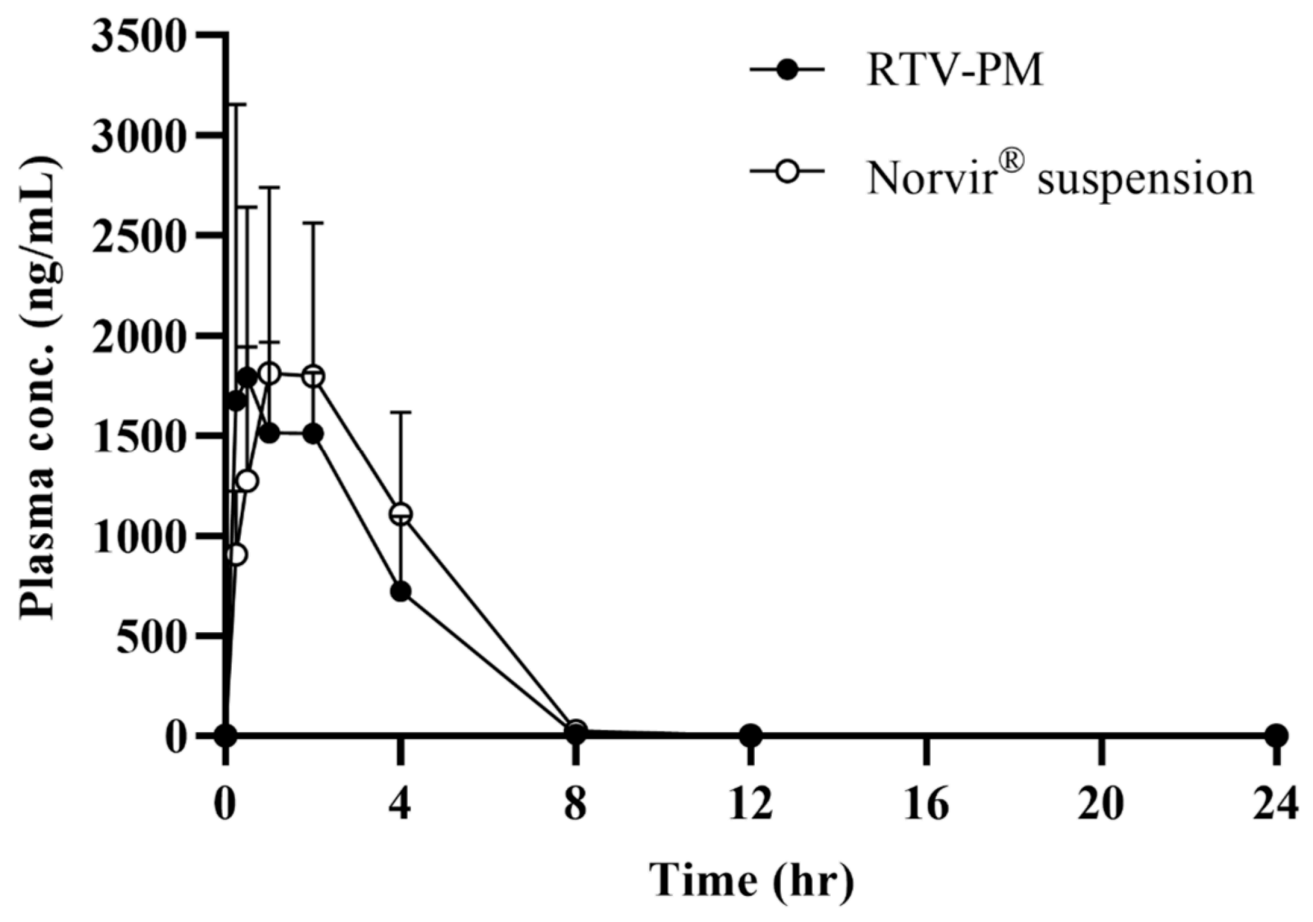
Mean plasma concentration of RTV in rats after oral administration of RTV-PM and Norvir^®^ tablet suspension (*n* = 6, mean ± SD).

The plasma concentration–time profiles of RTV are presented in Figure 6, and the corresponding pharmacokinetic parameters are summarized in Table 5. RTV-loaded polymeric micelles exhibited pharmacokinetic performance comparable to that of the commercial product (Norvir^®^). The two formulations showed similar peak plasma concentrations (C_max_, 2243.9 ± 1226.4 vs. 2106.1 ± 835.9 ng/mL) and similar systemic exposure (AUC_last_, 6695.3 ± 2162.6 vs. 8178.2 ± 2892.1 h·ng/mL), indicating comparable oral bioavailability. RTV-loaded polymeric micelles showed a shorter T_max_ (0.9 ± 0.6 vs. 1.5 ± 0.5 h) and a shorter half-life (0.9 ± 0.3 vs. 1.1 ± 0.5 h), suggesting more rapid absorption; however, none of these differences reached statistical significance. In both groups, plasma RTV concentrations declined rapidly and were near baseline by 8 h post-administration, consistent with the short half-life of RTV.

Polymeric micelles have previously been applied to improve the oral delivery of RTV. Mahajan et al. reported tocofersolan-based micelles prepared by thin-film hydration, which achieved a particle size of 93.81 nm and a drug loading content of 19.54 ± 1.53% [20]. The present work differs in three respects (Table S1). First, we employed PEG-*b*-PCL, a biodegradable amphiphilic block copolymer that self-assembles into a core–shell structure, rather than a surfactant-based micellar system; this yielded a smaller particle size (25.9 ± 0.09 nm) and a higher drug loading content (27.3%, *w*/*w*). Second, whereas the previous study evaluated the micelle formulation against an in-house RTV suspension, we compared the oral pharmacokinetics with those of the marketed tablet (Norvir^®^) and framed the benefit in terms of excipient minimization, increasing the drug content from 12.5% to 27.3% (*w*/*w*) and thereby reducing the excipients required per unit dose by approximately 2.6-fold. Third, the micelles retained their initial particle size and drug loading content after lyophilization and reconstitution without the addition of any cryoprotectant, whereas mannitol was required in the previous study; this avoids introducing further excipients and is consistent with our objective of minimizing the excipient burden in a solid oral dosage form.

This study has some limitations. Solid-state characterization by DSC and PXRD was not performed, and such analyses would further clarify the physical state of RTV within the micellar core. Zeta potential was not evaluated, as the colloidal stability of PEG-*b*-PCL micelles is governed primarily by steric stabilization from the PEG corona rather than by electrostatic repulsion; nevertheless, surface charge measurement would provide complementary information. The CMC of PEG_2k_-*b*-PCL*2k* was also not determined, which would provide further insight into micelle stability upon dilution. These aspects remain subjects for future work. In addition, although RTV is a marketed drug and both PEG and PCL are biocompatible, biodegradable polymers with established safety profiles, a systematic toxicity evaluation was not performed, and comprehensive toxicity and repeated-dose safety assessments of the PEG-*b*-PCL carrier will be required before further development. The LC-MS/MS method used for plasma quantification was not formally validated for accuracy, precision, recovery, and the matrix effect, which should be established in future studies. Furthermore, the pharmacokinetic evaluation was limited to a rodent model, and interspecies physiological differences may affect translational relevance to humans. This exploratory study (n = 6/group) also showed relatively high PK variability, warranting larger, adequately powered studies. Studies in higher-order species or a pilot clinical study would help validate these findings in a more clinically relevant setting. The short-term (4-week) storage stability study was also conducted under uncontrolled temperature and humidity without an evaluation of physical appearance, reconstitution time, or residual moisture, and longer-term studies under controlled conditions are needed to support extended storage claims.

## 3. Materials and Methods

### 3.1. Materials

RTV and Norvir^®^ were donated by Hanmi Pharmaceutical Co. (Hwasung, Republic of Korea). Dichloromethane, ε-caprolactone, and stannous octoate (Sn(Oct)_2_) were purchased from Sigma-Aldrich (St. Louis, MO, USA). Methoxy poly(ethylene glycol) (mPEG-OH) was purchased from Tokyo Chemical Industry Co. (Tokyo, Japan). All other chemicals were of reagents grade and were used without further purification.

### 3.2. Synthesis of PEG-b-PCL Copolymers

PEG-*b*-PCL copolymers were synthesized by the ROP of ε-caprolactone initiated by mPEG-OH in the presence of Sn(Oct)_2_ as a catalyst according to a previously reported method with slight modifications (Figure 1A) [39]. In brief, pre-weighed mPEG-OH, ε-caprolactone and Sn(Oct)_2_ were mixed in a pressure tube, and the reaction was carried out at 120 °C for 24 h with stirring under an argon atmosphere [39]. For purification, dichloromethane was added to dissolve the reaction mixture after cooling at room temperature, then precipitated into cold diethyl ether to remove any residual reactants, followed by dissolving in distilled water and lyophilization to yield a white powder. Their molecular structures and average molecular weights were determined by ^1^H NMR spectroscopy and GPC.

### 3.3. Preparation of RTV-Loaded Polymeric Micelles

RTV-loaded polymeric micelles were prepared by the thin-film hydration method [41,42]. Briefly, both RTV and polymer stock solutions were prepared in organic solvents at 5 mg/mL and were mixed at a pre-determined ratio (4:10 drug-to-polymer *w*/*w* ratio). The solvent was removed by rotary evaporation at 50 °C for 10 min to obtain a thin film of the RTV/polymer mixture. The resultant thin film was hydrated with 1 mL of distilled water and incubated at 50 °C for 5 min to induce self-assembly and obtain drug-loaded polymeric micelles. The micelle solution was filtered through 0.22 μm syringe filters to remove any remaining unloaded drug and lyophilized to obtain drug-loaded micelle powder.

### 3.4. Characterization of RTV-Loaded Polymeric Micelles

#### 3.4.1. Particle Size Distribution of Micelles

The mean particle size and PDI of RTV-loaded polymeric micelles were analyzed by DLS using ELSZ-2000 (Otsuka, Osaka, Japan). Samples were diluted with distilled water to a polymer concentration of 1 mg/mL before particle size analysis. Measurements were performed at 25.0 °C using water as the dispersant (refractive index 1.3328, viscosity 0.8878 cP), with a count rate of approximately 70,000 cps. For each sample, 50 measurements were acquired per run, and three replicate measurements were performed. Particle size distributions are reported as intensity-weighted distributions.

#### 3.4.2. Determination of Drug Content in Micelles

The drug concentration in RTV-loaded polymeric micelles was analyzed by HPLC (Agilent Technologies 1260 Infinity) using a ZORBAX 300SB-C18 column (4.6 mm × 150 mm, 5 μm; Agilent Technologies, Santa Clara, CA, USA) according to a previously reported method with slight modifications [43]. The mobile phase consisted of acetonitrile and distilled water (50:50 *v*/*v*). The samples were diluted with acetonitrile to a final concentration of 0.4 mg/mL of RTV, and 10 μL of the sample was injected into the HPLC system. The flow rate was 1.0 mL/min, and column temperature was 30 °C. The detection wavelength was 240 nm.

Encapsulation efficiency (EE) and drug loading (DL) were calculated by the following equations:

(1) $$EE(\%)=\frac{weight\;of\;drug\;in\;the\;micelles}{weight\;of\;the\;feeding\;drug}\;\times 100$$

(2) $$DL(\%)=\frac{weight\;of\;drug\;in\;the\;micelles}{weight\;of\;drug\;in\;micelle\;and\;polymer}\times 100$$

#### 3.4.3. Morphology Study

The morphology of RTV-loaded polymeric micelles was analyzed by TEM (HT-7700, Hitachi, Japan). A drop of micelle solution was placed on a copper grid coated with formvar/carbon film, stained with 1% sodium phosphotungstate solution and dried at room temperature before micelle morphology observation.

### 3.5. Stability to Lyophilization

Prepared RTV-loaded polymeric micelle solution was frozen at −80 °C over 12 h and kept in liquid nitrogen for 15 min, followed by lyophilization for 24 h. The lyophilized drug-loaded micelle powder was stored at room temperature on a laboratory shelf, without controlled temperature or humidity, for 4 weeks. At pre-determined time intervals, the aliquots of the lyophilized powder were redispersed in distilled water to reconstitute the RTV-loaded micelles. Particle size distribution and drug concentration were analyzed by DLS and HPLC, respectively, as described above.

### 3.6. In Vitro Drug Release Study

The release of RTV from the polymeric micelles was performed using the dialysis method. A total of 100 μL of micelle solutions was loaded into dialysis devices (Slide-A-Lyzer MINI Dialysis Devices, 3.5 kDa MWCO, Thermo Scientific Inc., Waltham, MA, USA) and floated in a glass beaker with 900 mL of pH 1.2 HCl buffer solution with 100 rpm agitation at 37 °C. The samples were taken from dialysis devices at pre-determined time intervals and lyophilized. The amount of RTV in the samples was analyzed by HPLC as described above. The release profile of the RTV released from the polymeric micelles was plotted as the percentage cumulative amount of drug released over time.

### 3.7. In Vivo Pharmacokinetic Study

All animal experiments were approved by the Institutional Animal Care and Use Committee at Kyungpook National University (Permit number: 2023-0511). Male Sprague-Dawley (SD) rats (200–250 g, 5 weeks old) were purchased from Samtako Co. (Osan, Republic of Korea) and allowed to acclimate for one week. All rats were randomly assigned to two groups (*n* = 6) and fasted for 24 h with free access to water prior to the administration. The control group was orally administered with 1 mL of Norvir^®^ tablet suspension, prepared by crushing Norvir^®^ tablets and suspending the resulting powder in distilled water immediately before administration, at a dose equivalent to 10 mg/kg of RTV, which was selected based on a previous rat pharmacokinetic study of RTV [44]. The experimental group was orally administered with 1 mL of RTV-loaded polymeric micelle solution (RTV-PM) at the same dose of RTV for the control group after being redispersed in distilled water. About 300 μL of blood samples was collected from the jugular vein using heparinized syringes at pre-determined time points (0, 0.25, 0.5, 1, 2, 4, 8, 12, and 24 h post-dose), followed by centrifugation at 13,000 rpm for 5 min. The supernatant plasma was stored at −80 °C until analysis by LC-MS/MS. Plasma samples were processed by protein precipitation. Briefly, 10 μL of acetonitrile was added to 90 μL of plasma, followed by 20 μL of internal standard (IS) solution (lopinavir, 1 μg/mL in acetonitrile) and 300 μL of acetonitrile. The mixture was vortexed for 3 min and centrifuged at 13,000 rpm for 10 min, and the supernatant was injected into the LC-MS/MS system.

Chromatographic separation was performed on a Kinetex C18 column (100 × 2.1 mm, 2.6 μm, Phenomenex, Torrance, CA, USA) using a Vanquish UHPLC system coupled to a TSQ Altis Plus triple quadrupole mass spectrometer (Thermo Fisher Scientific, Bremen, Germany). The mobile phase consisted of 0.1% formic acid in water (A) and 0.1% formic acid in acetonitrile (B), delivered at a flow rate of 0.3 mL/min with a total run time of 3 min. Detection was performed in positive selected reaction monitoring (SRM) mode with electrospray ionization at 3.5 kV. The following transitions were monitored: m/z 721.3 → 295.9 for RTV (collision energy −15 eV) and m/z 629.3 → 447.1 for lopinavir (IS, collision energy −19 eV). Calibration standards were prepared in acetonitrile, with a lower limit of quantification (LLOQ) of 2 ng/mL for RTV. Data were processed using Xcalibur software (version 4.1; Thermo Fisher Scientific, Bremen, Germany). No target signal was detected in zero-blank and double-blank samples, confirming the absence of interference at the retention time of the analyte.

### 3.8. Statistical Analysis

All data were expressed as the mean ± SD. Comparisons between groups were performed using Student’s *t*-test (two-tailed), with *p* < 0.05 considered statistically significant. The normality of the data distribution was not formally tested given the sample size, and the *t*-test was applied based on the assumption of approximate normality. C_max_ and AUC were log-transformed prior to analysis due to their typically skewed distribution. No data points were excluded as outliers. Statistical significance was determined using the T.TEST function in Microsoft Excel (Version 2608).

## 4. Conclusions

The current study explored the potential of polymeric micelles composed of synthesized PEG-*b*-PCL copolymers to address significant limitations in conventional RTV formulations, primarily focusing on achieving high drug loading and improving the oral bioavailability of the drug. By systematically varying the molecular weights of PEG and PCL blocks, we identified PEG_2k_-*b*-PCL_2k_ as the optimal copolymer due to its superior drug loading efficiency, uniform particle size distribution, and stable micellar morphology. Stability testing revealed that the RTV-loaded micelles maintained their physicochemical characteristics upon lyophilization and reconstitution, suggesting robustness and suitability for practical pharmaceutical applications. The in vitro drug release study demonstrated prolonged RTV release under the acidic in vitro conditions examined. Pharmacokinetic studies further supported these findings, showing comparable systemic exposure between the optimized RTV-loaded polymeric micelle formulation and the commercial product, Norvir^®^. Overall, PEG-*b*-PCL-based polymeric micelles substantially reduced the excipient burden of RTV while maintaining comparable oral exposure, supporting their potential to improve patient compliance in antiretroviral therapy.

## 5. Patents

A patent application related to this work was filed in the Republic of Korea (Patent Application No. 10-2025-0120145).

## Figures and Tables

**Table 1:** Molecular weight characteristics of PEG-*b*-PCL copolymers.

Block Copolymer	M_n_^1^	M_n_^2^	M_w_^3^	PDI ^4^
PEG_2k_-*b*-PCL_1.5k_	3312	5200	6352	1.22
PEG_2k_-*b*-PCL_2k_	3997	5580	5978	1.07
PEG_2k_-*b*-PCL_2.5k_	4454	6613	8114	1.23
PEG_4k_-*b*-PCL_1.5k_	5312	7326	7947	1.08
PEG_4k_-*b*-PCL_2k_	5712	7576	8358	1.10
PEG_4k_-*b*-PCL_2.5k_	6739	8151	9169	1.12

^1^ M_n_ determined by ^1^H NMR; ^2^ M_n_ determined by GPC; ^3^ M_w_ determined by GPC; ^4^ PDI determined by GPC.

**Table 2:** Mean particle size, PDI, encapsulation efficiency (EE) and drug loading (DL) of drug-loaded micelles prepared with different molecular weights of PEG-*b*-PCL (theoretical drug concentration in micelle solution = 2 mg/mL) (*n* = 3, mean ± SD).

Block Copolymer	Avg. Diameter (nm)	PDI	EE (%) ^1^	DL (%) ^2^
PEG_2k_-*b*-PCL_1.5k_	91.3 ± 2.24	0.250 ± 0.005	72.9 ± 0.61	20.8 ± 0.17
PEG_2k_-*b*-PCL_2k_	26.3 ± 0.19	0.130 ± 0.032	96.4 ± 0.79	27.5 ± 0.23
PEG_2k_-*b*-PCL_2.5k_	72.5 ± 0.36	0.272 ± 0.004	65.7 ± 6.72	18.8 ± 1.92
PEG_4k_-*b*-PCL_1.5k_	42.6 ± 0.59	0.276 ± 0.006	35.3 ± 0.80	10.1 ± 0.23
PEG_4k_-*b*-PCL_2k_	50.8 ± 0.75	0.285 ± 0.007	31.7 ± 0.65	9.1 ± 0.19
PEG_4k_-*b*-PCL_2.5k_	75.1 ± 0.69	0.275 ± 0.004	49.0 ± 1.32	14.0 ± 0.38

^1^ EE (%) = *M_drug_/(M_drug added_)* × 100; ^2^ DL (%) = *M_drug_/(M_drug_ + M_excipient_)* × 100.

**Table 3:** Particle size distribution, encapsulation efficiency (EE) and drug loading (DL) of drug-loaded polymeric micelles prepared in different solvents (theoretical drug concentration in micelle solution = 2 mg/mL) (*n* = 3, mean ± SD).

Organic Solvent	Avg. Diameter (nm)	PDI	EE (%)	DL (%)
Acetonitrile	37.4 ± 3.56	0.189 ± 0.055	90.8 ± 0.35	26.7 ± 0.08
Methanol	25.8 ± 0.99	0.212 ± 0.005	93.9 ± 3.08	27.3 ± 0.65
Ethanol	51.5 ± 0.29	0.245 ± 0.011	93.4 ± 2.60	27.2 ± 0.55

**Table 4:** Comparison of particle size distribution and drug loading content after reconstitution of lyophilized micelle solution (theoretical drug concentration in micelle solution = 2 mg/mL) (*n* = 3, mean ± SD).

Formulation	Avg. Diameter (nm)	PDI	Actual Drug Conc. (mg/mL)	EE (%)
Original micelle	25.9 ± 0.09	0.224 ± 0.008	1.98 ± 0.03	98.9 ± 1.54
Reconstituted micelle	24.4 ± 0.45	0.154 ± 0.012	2.00 ± 0.03	100.2 ± 1.48

**Table 5:** Pharmacokinetic parameters of RTV in rats after oral administration of RTV-PM and Norvir^®^ suspension (*n* = 6, mean ± SD).

Block Copolymer	RTV-PM	Norvir^®^ Suspension
t_1/2_ (h)	0.9 ± 0.3	1.1 ± 0.5
T_max_ (h)	0.9 ± 0.6	1.5 ± 0.5
C_max_ (ng/mL)	2243.9 ± 1226.4	2106.1 ± 835.9
AUC_last_ (h·ng/mL)	6695.3 ± 2162.6	8178.2 ± 2892.1
AUC_inf_ (h·ng/mL)	6700.8 ± 2159.7	8185.2 ± 2892.1

t_1/2_: half-time; T_max_: time of peak plasma concentration; C_max_: peak plasma concentration; AUC_last_: area under curve (from zero to last sampling time); AUC_inf_: area under curve (from zero to infinity).

## Data Availability

The original contributions presented in this study are included in the article/Supplementary Material. Further inquiries can be directed to the corresponding authors.

## Supplementary Materials

The following supporting information can be downloaded at: https://www.mdpi.com/article/10.3390/ph19091491/s1, Table S1: Comparison of the present RTV-loaded PEG-*b*-PCL polymeric micelle formulation with the previously reported tocofersolan-based micelle formulation.

## Abbreviations

The following abbreviations are used in this manuscript:

BCS	Biopharmaceutical Classification System
CMC	Critical Micelle Concentration
DL	Drug Loading
DLS	Dynamic Light Scattering
EE	Encapsulation Efficiency
GPC	Gel Permeation Chromatography
^1^H NMR	Proton Nuclear Magnetic Resonance
M_n_	Number-Average Molecular Weight
M_w_	Weight-Average Molecular Weight
PDI	Polydispersity Index
PEG-*b*-PCL	Poly(ethylene glycol)-*block*-poly(ε-caprolactone)
ROP	Ring-Opening Polymerization
RTV	Ritonavir
TEM	Transmission Electron Microscopy
