# Supplementary material for: Ritonavir-Loaded PEG-b-PCL Polymeric Micelles: Reduced Excipient Requirements and Comparable Oral Pharmacokinetics in Rats
Source: Pharmaceuticals (Basel). 2026 Sep 19;19(9):1491. doi: 10.3390/ph19091491 (PMC13610413; doi:10.3390/ph19091491)
Supplement: Supplementary file 1 [file pharmaceuticals-19-01491-s001.zip › pharmaceuticals-4530397-supplementary.pdf]

Supplementary Materials

Table S1. Comparison of the present RTV-loaded PEG-*b*-PCL polymeric micelle formulation with the previously reported tocofersolan-based micelle formulation.

**Parameter**	**This study**	**Mahajan et al. (2026)**
Carrier	PEG-*b*-PCL (biodegradable amphiphilic block copolymer)	Tocofersolan (TPGS-based surfactant)
Preparation method	Thin-film hydration	Thin-film hydration
Particle size (nm)	25.9 ± 0.09	93.81
Polydispersity index	0.224 ± 0.008	0.170
Encapsulation efficiency (%)	98.9 ± 1.54	97.76 ± 1.34
Drug loading content (%, w/w)	27.3	19.54 ± 1.53
Cryoprotectant for lyophilization	Not used	Mannitol, 3.0% (w/v)
Comparator in pharmacokinetic study	Marketed tablet (Norvir®)	In-house drug suspension
Animal model	Rat (male Sprague–Dawley)	Rabbit (New Zealand)
Focus of the comparison	Excipient minimization relative to the marketed product (drug content 12.5% → 27.3%, w/w)	Relative oral bioavailability versus a drug suspension

Values are presented as mean ± standard deviation where available. Data for Mahajan et al. are taken from the optimized batch (RV5) reported in that study [20]. Direct comparison of pharmacokinetic parameters between the two studies is not appropriate, as different animal species, doses, and comparator formulations were used.

PEG-b-PCL, poly(ethylene glycol)-block-poly(ε-caprolactone); RTV, ritonavir; TPGS, D-α-tocopheryl polyethylene glycol 1000 succinate.
